# Caries of the Alveolus

**Published:** 1894-09

**Authors:** 


ARTICLE VI.

### CARIES OF THE ALVEOLUS.

As a general rule dentists have not in the past given as much attention to pathological studies as they should. Our best men have been ambitious to shine as operators, or as mechanics, and have neglected the first and most important of all studies, if we are to be considered in any sense as medical men. Beautiful fillings are too often inserted in teeth that are not in a physiological state, or which are in relation with diseased tissues, and the consequences are sometimes very serious. Many teeth are extracted simply because an otherwise excellent operator is not skilled in diagnosis and treatment. Diseases which, if properly treated at the outset, might be easily cured, are not promptly recognized, and are temporarized with, receiving only topical applications, until the general surgeon must be called in to remedy the effects of the lack of knowledge. Serious tumors have been dallied with until they have invaded tissues which should have been saved from their ravages.

Many of our schools have not given the attention to surgical pathology that is its due. In some there is no real comprehensive course of lectures upon this subject, the usual diseases of the teeth themselves comprising the instruction in this department, surgery of the jaws and face being entirely relegated to the general practitioner. There is excuse for this in the fact that our curriculum is already so broad that it is difficult in a term of six months to find time for the other lectures and demonstrations. Yet the dentist certainly should be able to diagnose any diseased condition, even though he should desire to turn it over to a specialist.

But there are some conditions requiring surgical interference that should never be allowed to go out of the dentist's hands. One of these is caries of the alveolus. This is a disease that is far more common than the average dentist is aware of. By it is meant the death and disintegration of the alveolar portions of the bone, cell by cell and without any serious complications. It differs from necrosis in that there is not usually any formation of pus, or at least but little, no special tumefaction or inflammation of the soft tissues, and no tendency towards a sequestrum. In necrosis there is a stoppage of nutrition throughout a considerable portion of osseous tissue, while in caries it is a slowly progressing ostisis that breaks down the bone one cell at a time.

Not infrequently, in the filling of cavities in proximate surfaces of the teeth, which extend well up in the cervical region, the alveolus between them is injured by wedges, or matrices, or separators and this induces an ostitis which results in a caries that causes the loss of the whole of the septum. It is at best but a thin lamina, and hence is specially liable to loss of nutrition through injury. We have known cases in which true necrosis has been the result of such injuries, and a real sequestrum of bone has exfoliated.

Sometimes when there has been violent periostis about a tooth root, the inflammation has spread to the bone and a carious action been set up. In many instances this will continue after extraction. The cavity, perhaps will not fill up by organization of the plastic exude, the gum will not heal over, and a small cavity will be left. There will be little of inflammation or soreness, and the attention of the patient will not be called to it until the cavity has assumed considerable proportions. If, then, an examination be made with an exploring instrument, the bone will be found bare, and it will be soft and spongy. It will not feel precisely like necrotic bone, but the difference between this and sound tissue can be readily recognized.

A carious condition of the alveolus will sometimes be the result of an anæmic or atonic state. It may be indicative of an inherited syphilitic taint. Of course it will be most often found in those who are in a debilitated state. The dentist should always examine for this when such patients who have been subjected to dental operations fall into his hands. It may be that he will need to scrutinize closely, for sometimes it is not very manifest.

The treatment, of course, is carefully to remove that which is carious and spongy, apply antiseptics, these to be followed by stimulating applications, like iodide and chloride of zinc, ten to twenty grains to the ounce of water. If the caries is at all extensive, the patient will probably be in an atonic condition, and alteratives, with liberal diet and careful hygienic precautions should be prescribed. Preparations of iron may be administered, the hypophosphites are useful, and tonics generally. If there is reason to suspect any constitutional taint iodide of potassium may be prescribed, or the syrup of iodide of iron, from twenty to forty drops three times per day.

But if the dentist does not desire to attempt general remedies, at least he can and should remove the carious bone by means of bone curettes, or perhaps the engine bur, and give such tropical treatment as is needed. Unless he is competent to diagnose the condition and do this much, he can scarcely call himself a surgeon dentist.—*Dental Practitioner and Advertiser.*

---

